# Digital orthodontic radiographic set versus cone-beam computed tomography: an evaluation of the effective dose

**DOI:** 10.1590/2177-6709.21.4.066-072.oar

**Published:** 2016

**Authors:** Lillian Atsumi Simabuguro Chinem, Beatriz de Souza Vilella, Cláudia Lúcia de Pinho Maurício, Lucia Viviana Canevaro, Luiz Fernando Deluiz, Oswaldo de Vasconcellos Vilella

**Affiliations:** 1 Specialist in Orthodontics, Universidade Federal Fluminense (UFF), Niterói, Rio de Janeiro, Brazil.; 2 Professor, Graduate program in Orthodontics, Universidade Federal Fluminense (UFF), Niterói, Rio de Janeiro, Brazil.; 3 Professor, Graduate program in Radioprotection and Dosimetry, Instituto de Radioproteção e Dosimetria (IRD), Rio de Janeiro, Rio de Janeiro, Brazil.; 4 Professor, Graduate program in Radiology, Universidade Estácio de Sá (UNESA), Rio de Janeiro, Rio de Janeiro, Brazil.

**Keywords:** Cone-beam computed tomography, Digital radiograph, Radiation dose.

## Abstract

**Objective:**

The aim of this study was to compare the equivalent and effective doses of different digital radiographic methods (panoramic, lateral cephalometric and periapical) with cone-beam computed tomography (CBCT).

**Methods:**

Precalibrated thermoluminescent dosimeters were placed at 24 locations in an anthropomorphic phantom (Alderson Rando Phantom, Alderson Research Laboratories, New York, NY, USA), representing a medium sized adult. The following devices were tested: Heliodent Plus (Sirona Dental Systems, Bernsheim, Germany), Orthophos XG 5 (Sirona Dental Systems, Bernsheim, Germany) and i-CAT (Imaging Sciences International, Hatfield, PA, USA). The equivalent doses and effective doses were calculated considering the recommendations of the International Commission of Radiological Protection (ICRP) issued in 1990 and 2007.

**Results:**

Although the effective dose of the radiographic set corresponded to 17.5% (ICRP 1990) and 47.2% (ICRP 2007) of the CBCT dose, the equivalent doses of skin, bone surface and muscle obtained by the radiographic set were higher when compared to CBCT. However, in some areas, the radiation produced by the orthodontic set was higher due to the complete periapical examination.

**Conclusion:**

Considering the optimization principle of radiation protection, i-CAT tomography should be used only in specific and justified circumstances. Additionally, following the ALARA principle, single periapical radiographies covering restricted areas are more suitable than the complete periapical examination.

## RESUMO

**Objetivo:**

o objetivo deste estudo foi comparar as doses equivalentes e efetivas absorvidas pelo mesmo receptor, quando exposto a diferentes exames radiográficos digitais (panorâmica, telerradiografia lateral e periapicais) e à tomografia computadorizada de feixe cônico (TCFC).

**Métodos:**

dosímetros termoluminescentes pré-calibrados foram colocados em 24 locais em um *phantom* antropomórfico (Alderson Rando Phantom, Laboratórios de Pesquisas de Alderson, New York, NY, EUA), o que representa um adulto de tamanho médio. Os seguintes equipamentos foram avaliados: Heliodent Plus (Sirona Dental Systems, Bernsheim, Alemanha), Orthophos XG 5 (Sirona Dental Systems, Bernsheim, Alemanha) e i-CAT (Imaging Sciences International, Hatfield, PA, EUA). As doses equivalentes e as doses efetivas foram calculadas tendo-se em conta as recomendações da Comissão Internacional de Proteção Radiológica (*International Commission on Radiological Protection*, ICRP), publicadas em 1990 e 2007.

**Resultados:**

embora a dose eficaz do *set* radiográfico tenha correspondido a 17,5% (ICRP 1990) e 47,2% (ICRP 2007) da dose da TCFC, as doses equivalentes na pele e superfícies ósseas e musculares desse *set* foram maiores, quando comparadas às da TCFC. Em algumas áreas, a radiação produzida pelo *set* de radiografias foi maior, devido à radiação do exame periapical completo.

**Conclusão:**

considerando-se o princípio da otimização da proteção radiológica, a tomografia computadorizada adquirida no tomógrafo i-CAT deve ser usada apenas em circunstâncias específicas e justificadas. Além disso, seguindo o princípio ALARA, radiografias periapicais unitárias de áreas pré-selecionadas são mais apropriadas do que o exame periapical completo.

## INTRODUCTION

The limitation of radiography due to its two-dimensional representation of tridimensional structures is a well-known fact.^1^ ^,^ ^2^ In the last decades, two-dimensional images were gradually replaced by tridimensional ones. Cone-beam computed tomography (CBCT) provides a high-resolution image that is similar to computed tomography,^3^ but at lower cost and radiation dose.^4^ ^,^ ^5^ ^,^ ^6^ Given these advantages, CBCT use is widespread in Dentistry nowadays, particularly for diagnosis, treatment planning and follow-up.^7^

On the other hand, the high prevalence of adolescents who seek orthodontic treatment goes against the fact that the radiation emitted by CBCT is greater than the radiation emitted by a radiographic device. The higher frequency of young patients results in a concern regarding radiation dose, as children seem to carry the brunt of radiation for a longer period of time than adults, and their developing organs are more sensitive to radiation effects.^8^

Furthermore, due to stochastic effects, of which probability of occurring is proportional to the radiation dose without a threshold, limits had to be established. The ALARA principle is usually applied as a reference.^9^

In order to control the radiation doses emitted by the devices and to allow evaluations and comparisons of different devices, the International Commission of Radiological Protection (ICRP) established values in 1990 and 2007. These values were applied to calculate the absorbed dose, the equivalent and the effective dose. Although studies have already compared different models and parameters, significant differences between models and between imaging protocols of the same device were observed.^10^ ^,^ ^11^

The aim of this study is to compare the equivalent and effective doses of different digital radiographic methods (panoramic, lateral cephalometric and periapical) with cone-beam computed tomography (CBCT) absorbed by the same receptor.

## MATERIAL AND METHODS

### Calibration and selection of dosimeters

Two types of thermoluminescent dosimeters (TLD) were employed in this study: TLD-100 Chip (Thermo Fisher Scientific Incorporation, Waltham, MA, USA) and TLD-100 Rod (Thermo Fisher Scientific Incorporation, Waltham, MA, USA). Due to the attenuation of radiation by the tissues, chip dosimeters were positioned on skin areas while rods dosimeters, more sensitive, were adapted in the holes inside the phantom. TLDs were prepared, calibrated and evaluated following the routine procedures of the Thermoluminescent Dosimetry Laboratory of Instituto de Radioproteção e Dosimetria (IRD), Brazil. Thereafter, they were preselected in groups with similar sensitivities (standard deviation of the mean value were lower than 5%) after three Cs 137 free in air irradiations with electronic equilibrium. The air kerma values were of 1.0 mGy.

The selected dosimeters were wrapped in plastic, so as to protect them from dirt and moisture. Subsequently, each one of these plastic packs was placed in a specific location inside an Alderson Rando phantom in order to evaluate the organ/tissue equivalent dose. A total of 19 TLDs was chosen to measure the background dose.

### The location of dosimeters

The dosimeters were positioned in 24 regions (Table 1) of a phantom that was composed by the skeleton of a medium sized male adult (1.75 m) covered with equivalent tissue material. The areas were selected according to Ludlow's methods^6^ ^,^ ^12^ ^-^ ^15^ and corresponded to radiosensitive organs, including eyes and pituitary gland. All dosimeters were placed inside the phantom by the same operator in order to reduce positioning variability.

**Table 1:** Location of thermoluminescent dosimeters in Alderson Rando phantom.

Organ/Tissue	Location	TLDs
Bone marrow	Anterior calvarium	1
	Posterior calvarium	2
	Left calvarium	3
	Center cervical spine	12
	Right/left ramus	10, 11
	Right/left mandibular body	14, 15
Brain	Mid brain	7
	Pituitary fossa	4
Eyes	Right/left orbit	5, 6
	Right/left lens of eye	21-24*
Salivary glands	Right/left parotid	8, 9
	Right/left submandibular gland	16, 17
	Sublingual gland	13
Thyroid	Thyroid surface	19*
	Midline thyroid	18
Skin	Right cheek	25, 26*
	Left back of neck	27, 28*
Esophagus	Pharyngeal-esophageal space	20

*Dosimeters positioned on the surface of the phantom. TLDs: thermoluminescent dosimeters.

### Protocols and parameters adopted 

The devices evaluated were Heliodent Plus (Sirona Dental Systems, Bernsheim, Germany), Orthophos XG 5 (Sirona Dental Systems, Bernsheim, Germany) and CBCT i-CAT (Imaging Sciences International, Hatfield, Pa, USA). Protocols for digital radiograph and CBCT parameters for examination of a medium sized male adult were adopted (Table 2). For the periapical examination, exposure time varied according to the region; whereas for the CBCT examination a field of view (FOV) of 22 cm was necessary in order to obtain the image of all structures.

**Table 2:** Parameters adopted.

	FOV*	kV**	mA***	Exposure time (s)
Heliodent plus	Periapical	70	7	0.25 - 0.4
Orthophos XG 5	Panoramic	69	15	14.1
Orthophos XG 5	Cephalometric	80	14	9.4
i-CAT	22 cm	120	3 - 7	40

*Field of view. **Kilovoltage. *** Milliamperes.

The phantom was positioned according to the manufacturer's guidelines without the thyroid collar. Based on the doses obtained in other studies,^12^ ^,^ ^16^ and also to avoid underexposure and overexposure of the dosimeters, ten exposures for panoramic and lateral cephalometric radiographs, five exposures for periapical and one exposure for CBCT were performed.

### The equivalent and the effective doses

The values obtained were divided by the number of repeated irradiations, so as to obtain the value per examination.

For the bone marrow, the equivalent dose was calculated based on the distribution of bone throughout the adult body. The mandible contains 1.3%, the calvarium 11.8% and the cervical spine contains 3.4%. The technique by Underhill et al was adopted to calculate the dose for the calvarium. For bone surface, a correction factor was applied: 

Bone: muscle attenuation ratio = 



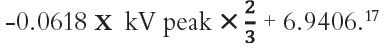



The proportion of skin area in the head and neck region directly exposed during maxillofacial CBCT imaging is estimated as 5% of the total body. Muscle and lymphatic nodes are estimated to represent 5%, esophageal tract 10% and other tissues 100%.^12^

The salivary glands began to be used in the effective dose calculation only in ICRP 2007.^18^ Their equivalent dose is obtained with the weighted average dose values of parotid, submandibular and sublingual glands.

The equivalent doses (H_T_) in these organ/tissues were calculated by the following formula:



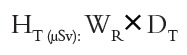



W_R_ is the radiation weighting factor and its value is 1 for X-rays. D_T_ is the mean absorbed dose in T.^19^

The effective dose (E), proposed by ICRP 1990,^18^ is a reliable clinical and standardized measure of the biological effects of radiation, although previous studies have demonstrated limitations.^20^ The effective dose defined to estimate an average whole body human radiation risk is calculated with the following formula: 

E = ∑w_T_ X H_T_ ^18^

W_T_ is the weighting factor of the organ or tissue (_T_) and is related to its radiation sensitivity. Both tissue weighting factors of ICRP 60 and ICRP 103 (1990 and 2007) applied in this study are presented in Table 3.

**Table 3:** Estimated percentage of tissue irradiated and dosimeters used to calculate mean absorbed dosage.

Organ/Tissue	Fraction irradiated (%)	TLD
Bone marrow	16.5	
Mandible	1.3	10, 11, 14, 15
Calvarium	11.8	1-3
Cervical spine	3.4	12
Thyroid	100	18, 19
Esophagus	10	20
Skin	5	21-28
Bone surface	16.5	
Mandible	1.3	10, 11, 14, 15
Calvarium	11.8	1-3
Cervical spine	3.4	12
Salivary glands	100	
Parotid	100	8, 9
Submandibular	100	16, 17
Sublingual	100	13
Brain*	100	4, 7
Remainder		
Brain**	100	4, 7
Lymphatic nodes*	5	1-12
Muscles*,**	5	1-12
Extrathoracic airway*	100	5, 6, 8-12
Oral mucosa*	100	8-11, 13

*ICRP 2007 recommendations , **ICRP 1990 recommendations. TLD: thermoluminescent dosimeter.

ICRP 103 (2007)^18^ increased the number of organs/tissues with w_T_ values, including brain and salivary glands, and the number of remainder tissues increased to 14. Only lymphatic nodes, muscles, extrathoracic airways and oral mucosa were exposed during the tests (Table 4).

**Table 4:** Tissue weighting factors - ICRP 1990 and 2007 recommendations.

Organ/Tissue	W_T_ 1990	W_T_2007
Bone marrow	0.12	0.12
Breast	0.05	0.12
Colon	0.12	0.12
Lung	0.12	0.12
Stomach	0.12	0.12
Bladder	0.05	0.04
Esophagus	0.05	0.05
Gonads	0.20	0.08
Liver	0.05	0.04
Thyroid	0.05	0.05
Bone surface	0.01	0.01
Brain	Remainder	0.01
Salivary glands	-	0.01
Skin	0.01	0.01
Remainder	0.05*	0.10**

*Adrenals, brain, upper large intestine, small intestine, kidney, muscle, pancreas, spleen, thymus and uterus. **Adrenals, extrathoracic region, gall bladder, heart, kidneys, lymphatic nodes, muscle, oral mucosa, pancreas, prostate, small intestine, spleen, thymus and uterus.

The new recommendations stated that brain and salivary glands received factors of 0.01 and 0.1, respectively. The oral mucosa equivalent dose was calculated by the salivary glands and mandibular ramus and body with a conservative estimate of 100%.^14^

## RESULTS

The obtained values of the equivalent and effective doses are listed in Table 5. The lowest equivalent doses were obtained in lateral cephalometric radiograph, followed by panoramic, periapical and CBCT. Considering thyroid equivalent doses, it was observed that their values were lower in lateral cephalometric and periapical examinations, and higher in CBCT. 

**Table 5:** Mean of equivalent doses (µSv) of each organ or tissue, effective doses (µSv) and percentage of equivalent and effective doses of all devices compared to CBCT.

Organ/Tissue	Cephalometric	Panoramic	Periapical	CBCT	Radiographic set	Radiographic set/CBCT (%)
Thyroid	5.1	34.5	1.1	388.5	40.7	10.5
Bone marrow	3.3	21.4	66.3	279.6	91	32.5
Esophagus	0.7	3.4	10	89.7	14.1	15.7
Skin	1	36.2	0.7	0.2	37.9	18950.0
Bone surface	12.1	87.7	3268.4	556.4	3368.2	605.4
Salivary glands	26.4	359.1	932.2	1908.2	1317.7	69.1
Brain**	14	33.9	139.7	2985.3	187.6	6.3
Remainder						
Brain*	14	33.9	139.7	2985.3	187.6	6.3
Lymphatic nodes**	1.3	18	46.6	95.4	65.9	69.1
Extrathoracic airways**	26.4	359.1	932.2	1908.2	1317.7	69.1
Muscles*,**	1	5.6	70.4	62.3	77	123.6
Oral mucosa**	23.2	316	839.7	1813.1	1178.9	65.0
Effective dosage ICRP 90	1.2	6.7	16.5	139.2	24.4	17.5
Effective dosage ICRP 07	2.5	27.1	69.1	208.9	98.7	47.2

*ICRP 1990; **ICRP 2007.

By adding salivary glands to the calculations of effective doses, their values increased considerably. The glands and the remainder tissues were the main contributors to the effective dose in lateral cephalometric and panoramic radiographs. The effective doses using values recommended by the ICRP 60 (1990)^19^ correspond to 48%, 24.7%, 23.8% and 66.6% of the doses calculated with the recommendations of the ICRP 103 (2007)^18^ for cephalometric, panoramic, periapical and CBCT, respectively. These results corroborate those of other studies.^6^ ^,^ ^12^

The equivalent and effective doses obtained by the radiographic set were summed up and the percentages between these values and the CBCT values were calculated (Table 5). Although the effective dose of the radiographic set corresponded to 17.5% (ICRP 1990) and 47.2% (ICRP 2007) of the CBCT dose, the equivalent doses of skin, bone surface and muscle obtained by the radiographic set were higher when compared to CBCT.

## DISCUSSION

In dosimetry, several factors must be considered: the phantom used (made from bones or just equivalent tissue material), number and location of dosimeters, type of device tested and its parameters (voltage [kV], amperage [mA], time of exposure, field of view [FOV] and voxel).^10^ Different combinations lead to different doses. Due to many variables, there are no appropriate parameters to compare these results, especially in relation to effective doses of radiographs and CBCT. Furthermore, in order to allow comparison between different studies, a standard methodology should be established.

A wide variation of effective doses was observed in different studies when evaluating CBCT scans. When comparing the same i-CAT model, different results were achieved.^6^ ^,^ ^12^ ^,^ ^15^ ^,^ ^21^ The high variability of radiation doses obtained compromises comparisons among different devices. 

Studies that evaluated current models, such as i-CAT Next Generation and i-CAT FLX, found effective doses of 182.1 µSv^15^ and 69.2 µSv,^21^ respectively. The reduction of FOV down to 17 cm and exposure time to 3.7 seconds may have contributed to reduce the effective dose, which reached about 38.9% when the results of these studies were compared with those of i-CAT FLX.

However, it was reported that the average distance between nasion and menton was 12.28 cm.^22^ While a multiethnic population presents much more variation, anterior facial height may reach greater values than these ones. Therefore, to the reduced field of view, even in the extended field of view protocol, some essential structures may be cut out of the image obtained.

The effective dose measured in i-CAT in the present study was greater than the sum of the effective doses of all radiographic examinations routinely required for orthodontic treatment. One reason for this difference might be the radiographic devices used in the study, which produce digital images with lower radiation doses. Additionally, i-CAT is a large volume tomographic device with extended FOV. The area exposed during examination is, therefore, increased.

The doses obtained in this study were higher in all devices because a lead apron was not used. The highest equivalent doses found were in the regions of the thyroid, brain and eyes. When a thyroid apron is used, there is a reduction of 48.7% in the dosage of the thyroid, and 41.7% in the dosage of the esophagus.^23^ Examinations with a large FOV showed a reduction of 61%.^24^ Therefore, the use of lead aprons should not be overlooked.

Additionally, the geometrical position of these organs in relation to the X-ray beam may have influenced the results. In i-CAT, due to the largest FOV, the organ is closer to the X-ray center of the beam.

The effective dose of the radiographic set corresponded to less than a half of the dose calculated for CBCT. On the other hand, the equivalent doses of skin, bone surface and muscle were higher (Table 5). The periapical examination was the most responsible for the highest dose. It could be due to proximity of dosimeters to the molars area. Therefore, to follow the ALARA^9^ principle (as low as possible radiation), the orthodontist should not request full periapical examination. Instead, single periapical radiographs covering restricted areas are more suitable.

Although the equivalent and effective doses of CBCT scans are high when compared to X-rays, the doses of multidetector CT scanners, used routinely for medical examinations, are dozens of times higher.^13^ ^,^ ^25^ Furthermore, it is estimated that the population is exposed to an average dose of natural radiation of 2400 µSv per year,^26^ and that the risk of developing cancer from exposure during CBCT examination is between 1:100.000 and 1:350.000 for adults.^19^

Moreover, tomography accepts the capture of a range of images otherwise inaccessible to radiography, whenever more in-depth information is needed about the patient. Nevertheless, based on the results of the current and other studies, CBCT examination with the i-CAT device should be indicated only in special cases and should not be used routinely.

## CONCLUSION

The effective doses produced by i-CAT were higher than the doses generated by the digital radiographs of the orthodontic set. However, in some areas, the radiation produced by the orthodontic set was higher due to complete periapical examination. Replacing radiographs with tomographic images generated by this device goes against the principle of ALARA and should be carried out only in specific cases. Furthermore, single periapical radiographs covering restricted areas are more suitable than complete periapical examination.
